# Global analysis of the association between pig muscle fatty acid composition and gene expression using RNA-Seq

**DOI:** 10.1038/s41598-022-27016-x

**Published:** 2023-01-11

**Authors:** Jesús Valdés-Hernández, Yuliaxis Ramayo-Caldas, Magí Passols, Cristina Sebastià, Lourdes Criado-Mesas, Daniel Crespo-Piazuelo, Anna Esteve-Codina, Anna Castelló, Armand Sánchez, Josep M. Folch

**Affiliations:** 1 Plant and Animal Genomics, Centre for Research in Agricultural Genomics (CRAG), CSIC-IRTA-UAB-UB, Campus UAB, Bellaterra, Spain; 2 Departament de Ciència Animal i dels Aliments, Facultat de Veterinària, Universitat Autònoma de Barcelona, Bellaterra, Spain; 3 Departament de Genètica i Millora Animal, Institut de Recerca y Tecnologia Agraroalimentàries (IRTA), Caldes de Montbui, Spain; 4 CNAG-CRG, Centre for Genomic Regulation, Barcelona Institute of Science and Technology, Barcelona, Spain

**Keywords:** Gene expression, Transcriptomics, Gene expression analysis, Gene expression profiling, Fatty acids

## Abstract

Fatty acids (FAs) play an essential role as mediators of cell signaling and signal transduction, affecting metabolic homeostasis and determining meat quality in pigs. However, FAs are transformed by the action of several genes, such as those encoding desaturases and elongases of FAs in lipogenic tissues. The aim of the current work was to identify candidate genes, biological processes, and pathways involved in the modulation of intramuscular FA profile from *longissimus dorsi* muscle. FA profile by gas chromatography of methyl esters and gene expression by RNA-Seq were determined in 129 Iberian × Duroc backcrossed pigs. An association analysis between the muscle transcriptome and its FA profile was performed, followed by a concordance and functional analysis. Overall, a list of well-known (e.g., *PLIN1*, *LEP, ELOVL6, SC5D*, *NCOA2*, *ACSL1, MDH1, LPL*, *LGALS12, TFRC, GOT1,* and *FBP1*) and novel (e.g., *TRARG1, TANK, ENSSSCG00000011196,* and *ENSSSCG00000038429*) candidate genes was identified, either in association with specific or several FA traits. Likewise, several of these genes belong to biological processes and pathways linked to energy, lipid, and carbohydrate metabolism, which seem determinants in the modulation of FA compositions. This study can contribute to elucidate the complex relationship between gene expression and FA profile in pig muscle.

## Introduction

Fatty acids (FAs) are crucial for living organisms, playing an essential role as mediators of signal transduction, cellular differentiation, and metabolic homeostasis. FAs can be classified into three groups (saturated: SFA, monounsaturated: MUFA, and polyunsaturated: PUFA) that are either provided by the diet or derived from de novo fatty acid (FA) synthesis^1,2^. FAs derived only from the diet are known as essential (e.g., C18:2*n*-6 and C18:3*n*-3) while those that can be synthesized through de novo lipogenesis are known as non-essential (e.g., C16:0, C16:1*n*-7, C18:0 and C18:1*n*-9)^3^.

Studies on pork meat quality and nutritive values have received special attention over the last decade. In fact, intramuscular FA content and its composition are important indicators of meat quality in pigs. In pork, meat quality parameters are mainly evaluated by changes in the flavour, oxidative stability, firmness, color^4,5^, and its nutritional value which is mainly determined by the FA profile. However, FA composition varies across tissues, and it is greatly affected by several factors such as environmental and host-factors including diet, fatness, body weight, gender, breed, and age, among others^6–8^. In addition, genetic background and gene expression are also able to influence the FA composition traits. In fact, the combination of methodologies such as gas chromatography and RNA sequencing (RNA-Seq), provides a powerful tool to analyze FA determinations^9^, as well as global changes in muscle transcriptome^10^, but also to discover genes contributing to intramuscular FA variation in lipogenic tissues from pig populations^11–13^.

The relationship between FA composition and gene expression is bidirectional. For example, dietary FAs like PUFA can affect gene expression by regulating the activity of several families of transcription factors (TFs, including PPARs, LXRs and SREBPs)^14^. By switching the direction of the effect, FA traits can also be modulated by genes encoding enzymes like desaturases and elongases in lipogenic tissues^2,15^. Notwithstanding, the regulatory mechanisms beyond these reactions are complex and involve the combination of TFs^2^, as well as several biological processes and pathways. In the context of animal and plant breeding, there is an increasing interest in identifying genes controlling the phenotypic variation of complex traits. That is the case of several studies focused on the genetic basis of intramuscular FA composition in pig muscle across several breeds^8,16–21^.

The aim of this work was to study the association between the porcine *longissimus dorsi* (LD) muscle FA profile and its transcriptome, focusing on the identification of the most relevant candidate genes, biological processes and pathways related to intramuscular FA composition.

## Methods

### Animals, sample collection and phenotypic data

A total of 129 animals generated by an experimental backcross named as BC1_DU (25% Iberian and 75% Duroc) were employed. All pigs were maintained under the same intensive conditions and fed ad libitum with a commercial cereal-based diet and free access to water. A more detailed description of the backcross BC1_LD generation, experimental design, animal raising, and feeding is provided in Martínez-Montes et al.^22^ Animal procedures were carried out according to the Spanish Policy for Animal Protection RD1201/05, which meets the European Union Directive 86/609 about the protection of animals used in experimentation. This study was conducted in accordance with relevant guidelines and regulations of the animal care and use committee of the Institut de Recerca i Tecnologia Agroalimentàries (IRTA), which adopts “The European Code of Conduct for Research Integrity” and “Good Experimental Practices”. Likewise, the experimental protocol was approved by the Ethical Committee of the IRTA. Our study is also reported in full compliance with ARRIVE guidelines (https://arriveguidelines.org/). Animals were slaughtered in five batches in a commercial abattoir of Mollerussa (Spain). Samples of *longissimus dorsi* (LD) muscle were collected, immediately snap frozen in liquid nitrogen and stored at − 80 °C until analysis. In addition, the distribution by sex was 59 females and 70 males, males were not castrated. At slaughter, pigs had an average age of 190 days (range 174–205 days), and 73.70 kg of carcass weight (range 46.10–109.20 kg).

FA composition in the C14-C22 range was determined using a gas chromatography of methyl esters protocol as described by Mach et al.^23^ in intramuscular LD muscle (n = 129). In brief, 200 g of LD muscle samples of 129 BC1_DU pigs were homogenized and used to measure the FA profile in duplicate. Additional information on the LD muscle FA composition in BC1_DU population is indicated in Crespo-Piazuelo et al.^20^.The FA composition (n = 17 FAs) was expressed as percentage of total identified FAs. Total percentages of SFA, MUFA, and PUFA were obtained through the sum of the individual FAs (Table 1). FA and metabolic ratios were calculated from the ratio between individual FA percentages as it is shown in Table 1. In addition, we further calculated the following FA metabolic indices:

$${\text{Average Chain Length }}\left( {{\text{ACL}}} \right) = \sum \left( {{\text{percentage of FAs }} \times {\text{ carbon length}}} \right),$$

$${\text{Double bond index }}\left( {{\text{DBI}}} \right) = \sum \left( {{\text{percentage of FAs }} \times {\text{ number of double bond}}} \right),$$

$${\text{Unsaturated Index }}\left( {{\text{UI}}} \right) = \left[ {\left( {{\text{DBI }} \times { 1}00} \right)/{\text{SFA}}} \right],{\text{ and}}$$

$$\begin{aligned} {\text{Peroxidability Index }}\left( {PI} \right) & = \sum \left[ {\left( {{\text{percentage of monoenoic acid }} \times \, 0.025} \right) \, + \, \left( {{\text{percentage of dienoic acid }} \times \, 1} \right)} \right. \\ \left. {\quad + \, \left( {{\text{percentage of trienoic acid }} \times \, 2} \right) \, + \, \left( {{\text{percentage of tetraenoic acid }} \times \, 4} \right)} \right]. \\ \end{aligned}$$

**Table 1:** Summary of descriptive statistics on the FA composition traits and FA metabolic indices in the LD muscle from BC1_DU pigs.

Trait	Name	Mean	SD	Min	Max	SEM	CV
C14:0	Myristic acid	1.27	0.23	0.73	1.78	0.02	17.95
C16:0	Palmitic acid	23.91	1.65	18.69	27.59	0.15	6.90
C17:0	Margaric acid	0.25	0.11	0.12	0.90	0.01	42.27
C18:0	Stearic acid	14.38	1.69	8.81	19.90	0.15	11.78
C20:0	Arachidic acid	0.23	0.08	0.08	0.71	0.01	33.94
C16:1*n*-7	Palmitoleic acid	2.79	0.53	1.24	4.08	0.05	18.83
C16:1*n*-9	9-Hexadecenoic acid	0.30	0.12	0.16	0.92	0.01	38.62
C17:1	Heptadecenoic acid	0.19	0.09	0.10	0.71	0.01	46.53
C18:1*n*-9	Oleic acid	35.93	5.71	19.99	44.15	0.50	15.88
C18:1*n*-7	Vaccenic acid	3.82	0.30	3.02	4.83	0.03	7.97
C20:1*n*-9	Gondoic acid	0.73	0.16	0.35	1.48	0.01	22.38
C18:2*n*-6	Linoleic acid	12.13	5.82	4.81	29.34	0.51	48.01
C18:3*n*-3	α-Linolenic acid	0.40	0.13	0.15	0.89	0.01	32.77
C20:2*n*-6	Eicosadienoic acid	0.43	0.12	0.14	0.91	0.01	28.61
C20:3*n*-3	Eicosatrienoic acid	0.18	0.10	0.02	0.65	0.01	54.98
C20:3*n*-6	Dihomo-gamma-linolenic acid	0.45	0.29	0.09	1.49	0.03	63.29
C20:4*n*-6	Arachidonic acid	2.58	1.94	0.47	10.51	0.17	75.01
SFA	Saturated FAs	40.04	3.05	29.11	46.11	0.27	7.61
MUFA	Monounsaturated FAs	43.47	6.13	26.17	52.30	0.54	14.11
PUFA	Polyunsaturated FAs	16.00	8.11	6.00	38.62	0.71	50.71
ACL	Average chain length	17.50	0.09	17.33	17.79	0.01	0.49
MUFA/SFA	Ratio of MUFA to SFA	1.09	0.13	0.62	1.36	0.01	11.84
MUFA/PUFA	Ratio of MUFA to PUFA	3.54	1.85	0.71	8.54	0.16	52.32
PUFA/SFA	Ratio of PUFA to SFA	0.42	0.25	0.14	1.24	0.02	61.27
C16:1*n*-7/C16:0	Ratio of palmitoleic to palmitic	0.12	0.02	0.05	0.16	0.001	16.11
C18:1*n*-9/C18:0	Ratio of oleic to stearic	2.51	0.37	1.22	3.39	0.03	14.87
C20:1*n*-9/C20:0	Ratio of gondoic to arachidic	3.49	1.30	1.07	10.84	0.11	37.30
C20:4*n*-6/C20:3*n*-6	Ratio of arachidonic to dihomo-gamma-linolenic	5.37	1.11	2.62	8.66	0.10	20.65
C18:1*n*-7/C16:1*n*-7	Ratio of vaccenic to palmitoleic	1.41	0.29	1.02	3.37	0.03	20.46
C20:3*n*-6/C18:2*n*-6	Ratio of dihomo-gamma-linolenic to linoleic	0.04	0.01	0.02	0.08	0.0007	25.52
C20:4*n*-6/C18:2*n*6	Ratio of arachidonic to linoleic	0.19	0.06	0.06	0.41	0.01	33.07
C18:2*n*-6/C18:3*n*-3	Ratio of linoleic to α-linolenic	30.23	10.25	12.15	73.65	0.90	33.92
ω6/ω3	Ratio of omega-6 to omega-3	26.46	8.07	14.33	49.78	0.71	30.48
PI	Peroxidability index	26.06	14.19	9.48	73.21	1.25	54.44
DBI	Double-bond index	0.82	0.15	0.63	1.31	0.01	18.14
UI	Unsaturated index	2.10	0.58	1.39	4.18	0.05	27.72

Fatty acid composition is expressed as percentage of total fatty acids. SEM—standard error of the mean. CV—coefficient of variation (in percentage). The following sums of fatty acids, ratios and indexes were calculated: SFA = C14:0 + C16:0 + C17:0 + C18:0 + C20:0; MUFA = C16:1*n*-7 + C17:1 + C18:1*n*-7 + C18:1*n*-9 + C20:1*n*-9; PUFA = C18:2n-6 + C18:3n-3 + C20:2n-6 + C20:3n-6 + C20:4n-6; ω6/ω3 = (C18:2*n*-6 + C20:2*n*-6 + C20:3*n*-6 + C20:4*n*-6)/(C18:3*n*-3 + C20:3*n*-3); Average Chain Length (ACL) = (C14:0) × 14 + (C16:0 + C16:1*n*-9 + C16:1*n*-7) × 16 + (C17:0 + C17:1) × 17 + (C18:0 + C18:1*n*-9 + C18:1*n*-7 + C18:2*n*-6 + C18:3*n*-3) × 18 + (C20:0 + C20:1*n*-9 + C20:2*n*-6 + C20:3*n*-6 + C20:4*n*-6 + C20:3*n*-3) × 20)/100; Double Bond Index (DBI) = (C16:1*n*-9 + C16:1*n*-7 + C17:1 + C18:1*n*-9 + C18:1*n*-7 + C20:1*n*-9) × 1 + (C18:2*n*-6 + C20:2*n*-6) × 2 + (C18:3*n*-3 + C20:3*n*-6 + C20:3*n*-3) × 3 + (C20:4*n*-6) × 4)/100; and Peroxidability Index (PI) = (C16:1*n*-9 + C16:1*n*-7 + C17:1 + C18:1*n*-9 + C18:1*n*-7 + C20:1*n*-9) × 0.025 + (C18:2*n*-6 + C202*n*-6) × 1 + (C18:3*n*-3 + C20:3*n*-6 + C20:3*n*-3) × 2 + (C20:4*n*-6) × 4.

### Total RNA isolation and sequencing

Total RNA was isolated from the LD muscle (100 mg) of 129 animals using the the RiboPure™ Isolation kit for High Quality Total RNA (Ambion^®^; Austin, TX) following the manufacturer’s recommendations. RNA quantification and purity was done with a NanoDrop ND-1000 spectrophotometer (NanoDrop products, Wilmington, DE, USA). RNA integrity was checked by Agilent Bioanalyzer-2100 equipment (Agilent Technologies, Inc., Santa Clara, CA, USA), using only those samples with an RNA integrity number (RIN) greater than 7 for the RNA-Seq experiment.

Library preparation and sequencing was performed at CNAG institute (Centro Nacional de Análisis Genómico, Barcelona, Spain). For each sample, one paired-end library was prepared using TruSeq Stranded mRNA kit (Illumina, Inc.; San Diego CA, USA). To discriminate among samples, libraries were labeled by barcoding and pooled to be run in Illumina HiSeq 3000/4000 instruments (Illumina, Inc.; San Diego CA, USA). In brief, in this study 2 × 75 bp reads, a mean of 45.09 million of paired-reads per sample, and an average of 90.06% (ranging from 80.51 to 96.09%) of uniquely mapped reads were generated.

### Bioinformatic analyses

Quality control and basic statistics of reads were performed using FastQC (v0.11.9)^24^ and MultiQC (v0.7)^25^ programs, respectively. Sequencing reads were mapped employing the STAR software (v2.7.9a) with default parameters^26^, and using the Sscrofa11.1 pig genome assembly as reference. Then, gene expression was quantified using RSEM (v1.2.28)^27^ software with default parameters and annotation from pig Ensembl Genes 97. Filtering was performed to keep rows of those genes with at least 129 reads in total, thus, 14,870 genes were retained. Normalization of the read counts was done as indicated below.

### Association between whole-transcriptome and FA profile in muscle

The association study was conducted using the ELMSeq approach^28^ on R v4.1.0^29^. We used the type I penalty function to test the association of gene expression with FA profile in LD muscle, while adjusting for all other covariates. The final model is described as follows:

1 $${\text{Trait}}_{i} = \beta_{0} + \beta_{1} {\text{Expression}}_{1i} + \beta_{2} {\text{Sex}}_{2i} + \beta_{3} {\text{Batch}}_{3i} + \varepsilon_{i}$$

where Trait_*i*_ represents each FA trait or FA index (n = 36) transformed into log_2_ base, *i* represents the individuals, β_0_ is the intercept, Expression_*i*_ indicates the normalized gene expression value [log(counts + log.add)], here "counts" is a matrix of *m* × *n* sizes, where *m* is the number of genes and *n* is the number of samples (14,870 × 129). β_1,_ β_2_ and β_3_ are the beta coefficients, respectively. Sex_*i*_ and slaughterhouse Batch_*i*_ represents fixed effects with 2 (female and male) and 5 levels (B1, B2, B3, B4 and B5), respectively. *ε*_*i*_ is the vector of residual effects. For ELMSeq, we used the sig_leval = 0.01, percentile = sig_leval, rho = 1, and log.add = 1e−3*min(counts[counts > 0]) parameters. Benjamini and Hochberg procedure^30^ was used to correct the raw *P*-value (BH adjusted *P*-value < 0.05) in the post-processing stage.

Among the total associated genes with each trait, we counted the number of lipid related genes using a gene list manually curated and compiled by our research group based on gene functional annotation accordingly to GO and pathway terms available in gene ontology databases (e.g., KEGG, Reactome, WikiPathways, STRING-db, AmiGO 2 and BioSystems of NCBI). The BioMart web tool (https://www.ensembl.org/) was used to extract attributes from Ensembl genome browser: (i) gene name, and (ii) human orthologues genes for those pig gene stable IDs without gene name. Afterwards, gene names were submitted (gene set augmentation) to the Geneshot tool using the ARCHS4 resource and the AutoRIF parameter^31^. Likewise, we compared the list of genes compiled in pig against the list of transcription factors (TFs) and TF co-factors from the AnimalTFDB v3.0 database (http://bioinfo.life.hust.edu.cn/AnimalTFDB/#!/).

### Concordance analysis

In order to have a preliminary quality control based on the biological role of the results of gene expression, a study of overlap between the number of associated genes with each trait was carried out. This analysis was organized by grouping the FA into five categories: (i) saturated FAs, (ii) monounsaturated FAs, (iii) polyunsaturated FAs, (iv) FA ratios, and (v) metabolic ratios and indices. Within each group, we explored the number of genes detected by each trait as well as the intersection size using the ComplexUpset package v1.3.3^32^.

### Gene ontology enrichment analysis

Gene Ontology (GO) enrichment analysis with the list of associated genes (by category among the trait class) were implemented using the ClueGO v2.5.8 plugin^33^ in Cytoscape v3.9.0 software^34^. All genes expressed in LD muscle were employed as background in the overrepresentation analysis across biological processes, molecular functions and pathways (KEGG). The statistical significance was assessed with a hypergeometric test using Benjamini and Hochberg method^30^ for multiple testing correction (BH corrected *P*-value < 0.05). In addition, a minimum k-score of 0.44 was used, GO tree interval levels set from three to eight, and a minimum of three genes per cluster with at least 4% in selected genes. Results with and without the fusion feature “GO Term Fusion” were generated to evaluate the redundant parent–child terms. To this end, ClueGo output was visualized using an R script that allowed to facet by categories via the ggplot2 package v3.3.5^35^.

### Correlation analysis

Pearson correlation analysis was carried out between the gene expression values and the FA phenotypic values using the R cor.test function. Here, the same normalization scale for the variables to be correlated was applied, as it has been described in the previous step of the ELMSeq algorithm. The Benjamini–Hochberg procedure^30^ was used to correct the raw *P*-value corresponding to the correlation coefficients. The goal of this strategy was to provide information about the direction of the association reported by ELMSeq, and therefore to obtain interpretable results regarding positive or negative relationships between the FA composition and the gene expression.

### Selection of candidate genes associated with the FA profile in muscle

The list of detected candidate genes were prioritized based on the following criteria: (i) candidate genes delimited into functional categories across biological processes, molecular functions and pathways (i.e., lipid metabolism, carbohydrate metabolism, amino acid metabolism, and nucleic acid metabolism terms); (ii) genes that correlate both specifically and simultaneously with FA phenotypes; and (iii) literature mining plus novelty assessment via Geneshot (including: rare, uncommon, common and very common levels).

## Results

### Identification of candidate associated genes with FA profile in muscle

In the first step we performed an association analysis using the ELMSeq algorithm to identify genes (n = 14,870 genes) associated with the profile of 36 FA in 129 BC1_DU pigs. We observed a variable number of associated genes across FA (1022 genes in total, Supplementary Table S1). Among the 36 traits, 22 FA were associated with a range of 1–553 genes. For each trait, we estimated the distribution of the number of associated genes across metabolic processes, lipid metabolism, TFs or co-factors, and novel genes (including long non-coding RNAs) (Supplementary Table S2).

**Figure 1:**
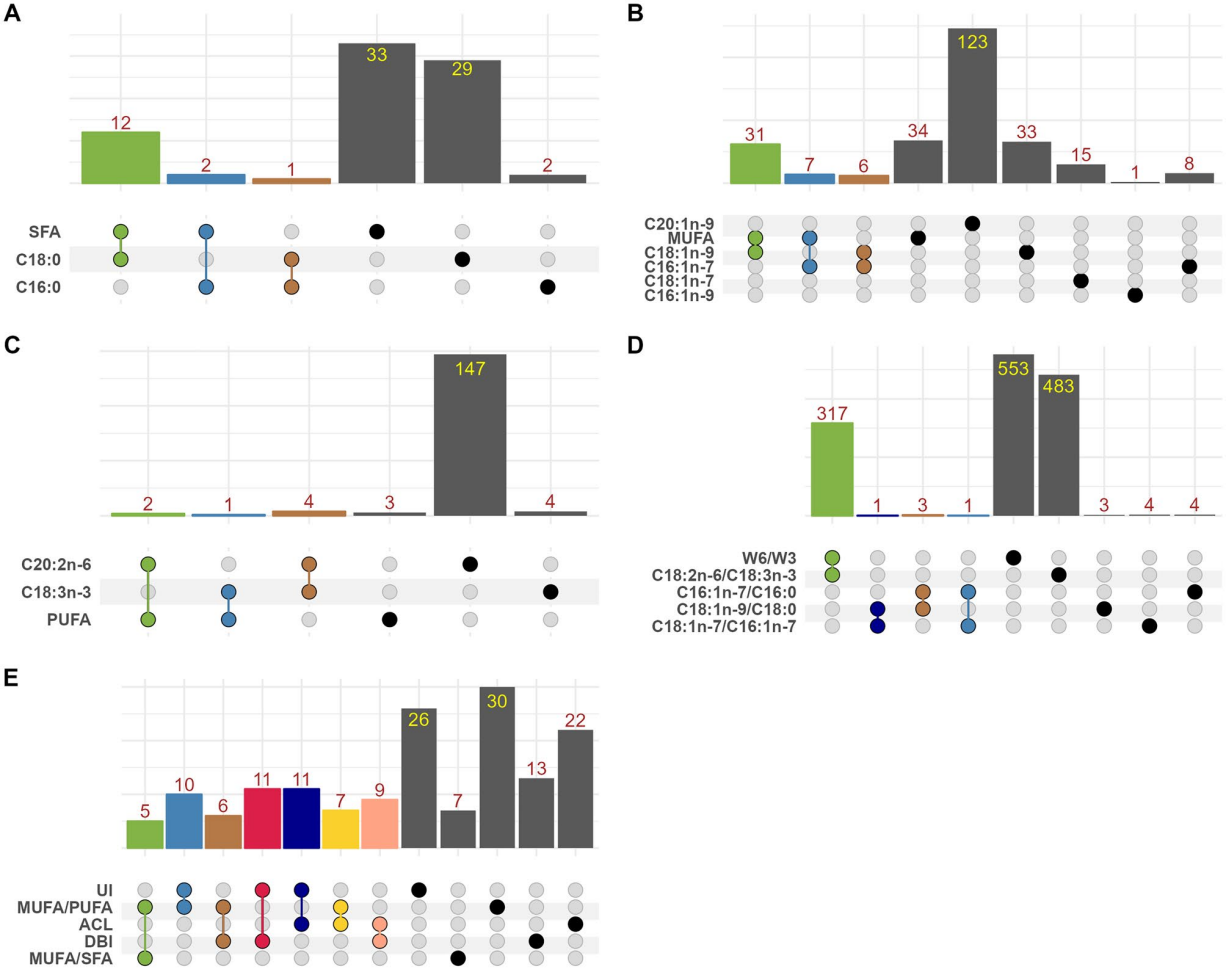
Analysis of overlapping and total number of associated genes by category of traits. The upset diagram (from left to right) shows the shared fraction (intersection size) of associated genes per pair of traits (colored bars), as well as the number of associated genes in each trait (gray bars). (**A**), (**B**), (**C**), (**D**) and (**E**) represents the group of saturated FAs, monounsaturated FAs, polyunsaturated FAs, FA ratios, and metabolic ratios and indices, respectively.

As shown in Fig. 1, it summarizes a description of the results corresponding to each of the FA categories: (1) saturated FAs, represented by three traits (C16:0, C18:0, and total SFA) with 2, 29 and 33 associated genes, respectively; (2) monounsaturated FAs, six traits (C16:1*n*-7, C18:1*n*-9, C18:1*n*-7, and C20:1*n*-9, C16:1*n*-9 and total MUFA) were linked to 8, 33, 15, 123, 1 and 34 associated genes, respectively; (3) polyunsaturated FAs, which includes two essential FAs (C18:3*n*-3 and C20:2*n*-6) and the total PUFA being associated to 4, 147 and 3 genes; (4) FA ratios (C16:1*n*-7/C16:0, C18:1*n*-7/C16:1*n*-7, C18:1*n*-9/C18:0, C18:2*n*-6/C18:3*n*-3, and ω6/ω3) linked to 4, 4, 3, 483 and 553 genes. Finally, on metabolic ratios and indices, five traits (MUFA/PUFA, MUFA/SFA, ACL, DBI, and UI) were linked with 30, 7, 22, 13 and 26 genes, respectively.

### Concordance analysis

The concordance analysis across the 22 FAs revealed that among the saturated FAs, 36.36% and 100% of the genes associated to C18:0 and C16:0 were commonly linked to the total SFA (Fig. 1A). On the other hand, monounsaturated FAs showed an overlap of genes associated to C16:1*n*-7 and C18:1*n*-9 compared with total MUFA (20.58% and 91.17%, respectively), and 18.18% of shared genes was found between C16:1*n*-7 and C18:1*n*-9 FAs (Fig. 1B). Regarding the polyunsaturated FAs, there were 33.33% and 66.66% overlapping genes between C18:3*n*-3, C20:2*n*-6 and total PUFA (Fig. 1C). Finally, the ω6/ω3 and C18:2*n*-6/C18:3*n*-3 ratios showed a 57.32% of common genes because the ω6/ω3 ratio contains C18:2*n*-6 and C18:3*n*-3 as major FAs, whereas the contrast between the rest of ratios showed a lower number of common genes (Fig. 1D).

### Gene ontology enrichment analysis

**Figure 2:**
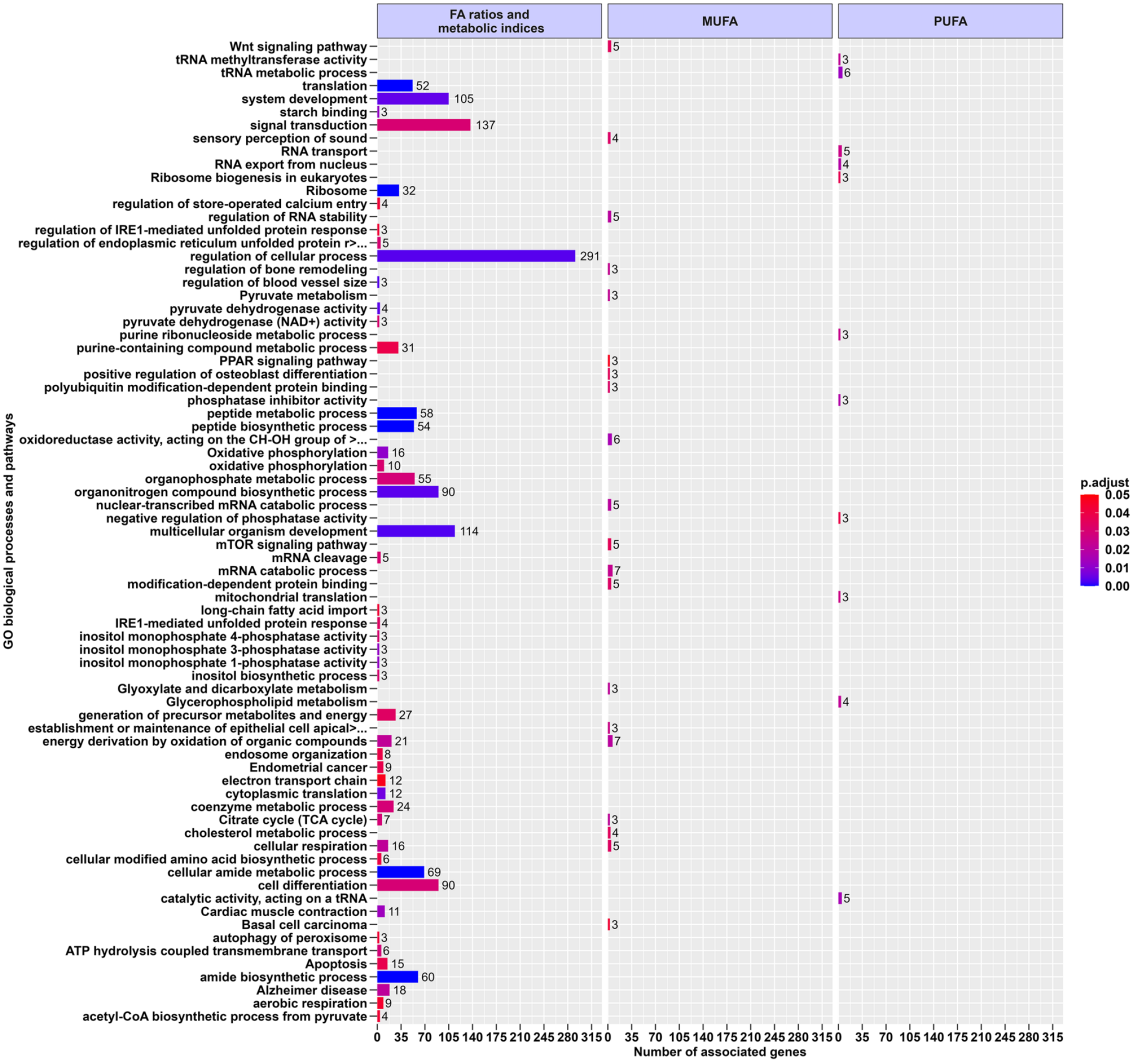
Functional enrichment analysis of the associated genes with FA profile and grouping by traits in BC1_DU pigs. The barplot with facets (from left to right) shows the enriched GO terms (BH adjusted *P*-value < 0.05) when grouping by traits. The traits grouped in this analysis are displayed in Fig. 1A–E. Legend: The full names to symbols " > …" are regulation of endoplasmic reticulum unfolded protein response; oxidoreductase activity, acting on the CH-OH group of donors, NAD or NADP as acceptor and establishment or maintenance of epithelial cell apical/basal polarity. GO terms of each individual category are presented in Table S3.

In the functional analysis, no significant GO terms/pathways were found for the list of associated genes (i.e., 50 in total) with the SFA category. Instead, for the other categories a total of 75 terms (biological process, molecular functions and pathways) were significantly enriched (Fig. 2). The full results (including genes by each GO terms) with and without “GO Term Fusion” can be consulted in Supplementary Table S3. As shown in Fig. 2, terms related to lipid metabolism were found on some categories, for example, “KEGG:03320 PPAR signaling pathway”, “GO:0008203 cholesterol metabolic process”, “KEGG:00564 Glycerophospholipid metabolism”, and “GO:0044539 long-chain fatty acid import”. Moreover, GO terms involved in carbohydrate metabolism were also enriched such as “GO:0006086 acetyl-CoA biosynthetic process from pyruvate”, “GO:0004738 pyruvate dehydrogenase activity, “KEGG:00020 Citrate cycle (TCA cycle)”, and “KEGG:00,620 Pyruvate metabolism”. Other GO terms were found related to carbohydrate and lipid homeostasis “KEGG:04310 Wnt signaling pathway” and control metabolic processes “KEGG:04150 mTOR signaling pathway”, respectively. In addition, GO terms related to some biological process, such as “GO:0015980 energy derivation by oxidation of organic compounds”, “GO:0006091 generation of precursor metabolites and energy”, “KEGG:00190 Oxidative phosphorylation”, “GO:2001256 regulation of store-operated calcium entry”, and “GO:0032543 mitochondrial translation”, were also enriched (Fig. 2). Finally, due to the low number of GO terms (n = 2, GO:0050880 and GO:0035150) found for the different metabolic ratios and indices, they were grouped into the single category term of “FA ratios and metabolic indices” (Fig. 2).

### Correlation analysis with candidate genes and representative traits

**Figure 3:**
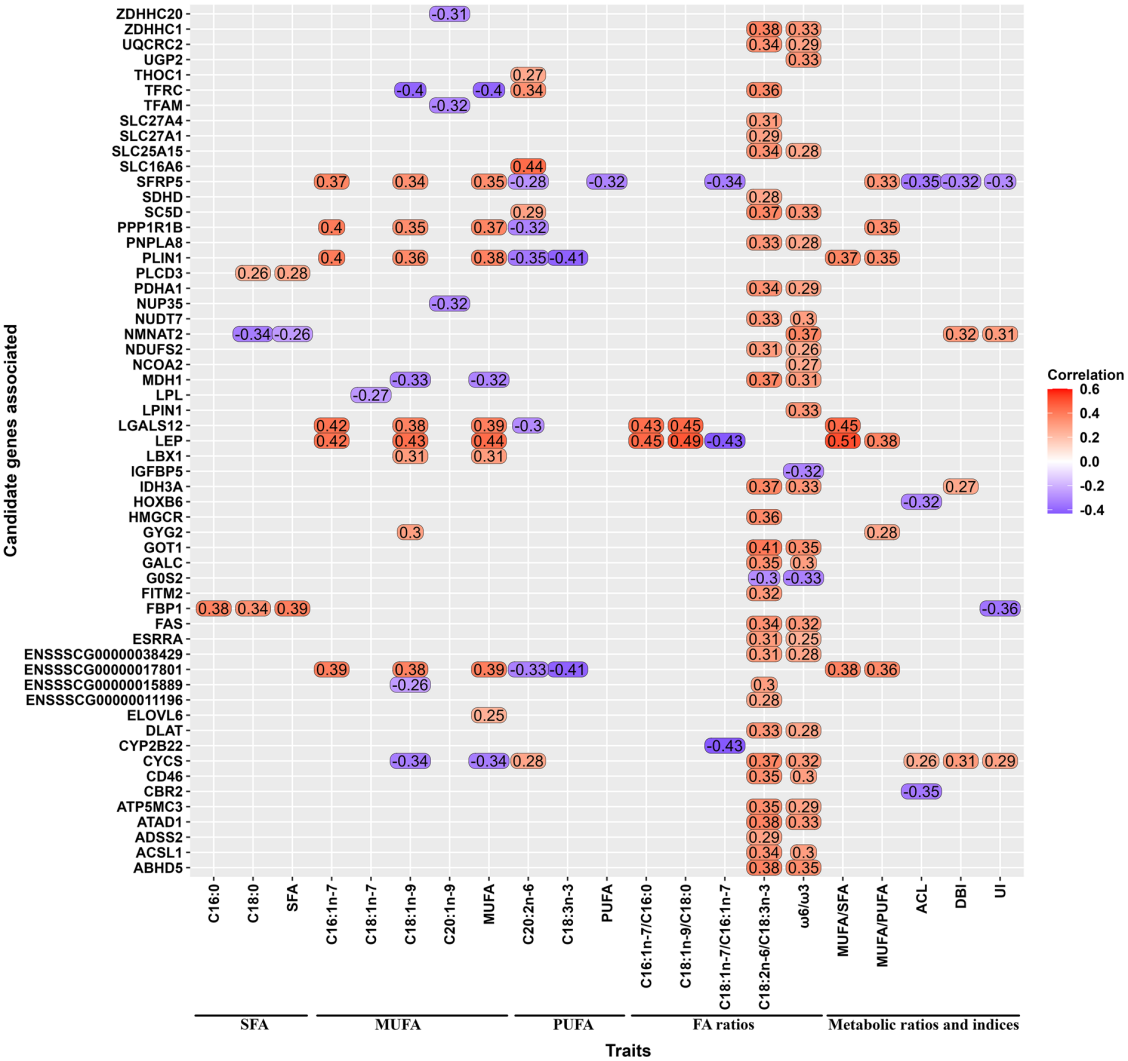
Heatmap of Pearson correlations of 21 representative traits with gene expression of 57 candidate genes related to FA profile traits in BC1_DU pigs. All these genes passed the correlation criteria according to a BH adjusted *P*-value < 0.05. The grouping of the FA profile traits is the same as that of the ELMSeq step.

Pearson correlations between the 22 FAs and their associated genes were calculated. Among these traits, 21 (excluding the C16:1*n*-9 FA) showed significant correlations (BH adjusted *P*-value < 0.05) with 547 genes (Supplementary Table S4). Out of these, 57 genes were prioritized by our analytical approach (Fig. 3). In general, the absolute correlation between gene expression and FA profiles ranged from low to moderate (from − 0.19 to 0.51). Details about particular correlations can be found in Fig. 3. We noticed several genes simultaneously correlated with more than one trait (*FBP1, PLIN1, MDH1, LEP, ACSL1, CYCS, SFRP5, PPP1R1B, IDH3A, TFRC, GOT1, SC5D, G0S2, PDHA1, LBX1, LGALS12, UQCRC2, PNPLA8, ABHD5, ESRRA, GYG2, ZDHHC1, PLCD3, UNC93A*, *ENSSSCG00000017801* alias *TRARG1, ENSSSCG00000015889* alias *TANK,* and *ENSSSCG00000038429*). We also observed genes specifically correlated with a single trait, such as: *LPL, ELOVL6, LPIN1, NCOA2, SDHD, SLC27A4, SLC16A6, SLC27A1, THOC1, TFAM, CYP2B22, ZDHHC20, NUP35, HOXB6, CBR2* and *IGFBP5*). Remarkably, when we explored the existence of genes inversely correlated between traits, we observed: (1) genes positively correlated with MUFA but negatively correlated with PUFA (*PLIN1, SFRP5, PPP1R1B,* and *TRARG1*), and vice versa (*CYCS* and *TFRC*); (2) genes positively correlated with MUFA and metabolic indices but negatively correlated with FA ratios (*LEP*); (3) genes positively correlated with MUFA and FA ratios but negatively correlated with PUFA (*LGALS12*); (4) genes negatively correlated with SFA but positively correlated with FA ratios and/or metabolic indices (*NMNAT2*); and (5) genes positively correlated with SFA traits but negatively correlated with metabolic indices (*FBP1*).

## Discussion

Studies on pork meat nutritive values and quality have received special attention over the last decade. Among other factors, meat nutritive value is determined by FA composition, playing also an important role in meat quality traits. Likewise, gene expression is assumed to be able to control the variation of such traits. In the present work, we aimed to study the association of muscle transcriptome with intramuscular FA profile from LD muscle to identify candidate genes, biological processes and pathways related with FA composition in pigs. Here, we used the ELMSeq approach^28^, which has been extensively employed in the study of human diseases like cancer or metabolic disorders. However, to the best of our knowledge, this is the first study that reports the use of ELMSeq approach to test the association between muscle transcriptome and FA composition in pigs or other livestock species.

Here, we reported well-known candidate genes, but also promising novel genes associated to the intramuscular FA profile (Supplementary Tables S1 and S2). In order to prioritize genes, the list of candidates was processed through an analytical pipeline including: (i) concordance analysis between the number of associated genes across categories of FAs (Fig. 1), (ii) ontology-based functional classification (Fig. 2 and Supplementary Table S3), and (iii) correlation analysis of FA phenotypes and respective gene expression of the associated genes (Fig. 3). As results, we highlighted 57 candidate genes including protein-coding, transcriptional regulators, and long-non coding RNAs (Fig. 3). In the following sections, we discuss some of these genes accordingly to their biological function, as well as the novelty of our findings (Supplementary Table S2).

### Genes related to biosynthesis and degradation of FAs

FAs are transformed by the actions of a vast array of enzymes and, we found a subset of genes that encode enzymes involved in the degradation and synthesis of FAs. This list of candidate genes included enzymes with lipolytic or lipase activity (*FBP1, LPL, MDH1*, *ACSL1,* and *GOT1*), and lipogenic activity (*ELOVL6*, *HMGCR* and *SC5D*). A detailed examination of the association study revealed that fructose-bisphosphatase 1 (*FBP1*) showed a synergistic effect (positively associated) with C16:0, C18:0 and SFA traits, but antagonistic (negatively associated) effect with UI trait (Fig. 3). *FBP1* catalyzes the hydrolysis of fructose 1,6-bisphosphate to fructose 6-phosphate and inorganic phosphate, acting as a gluconeogenesis regulatory enzyme (GeneCards ID: GC09M094603)^36^. The expression of ELOVL fatty acid elongase 6 (*ELOVL6*) was highly associated with MUFA (Fig. 3). *ELOVL6* encodes an enzyme responsible for condensation reaction, one of the four-step process (condensation, reduction, dehydration, and one further reduction) for elongation of very-long-chain (*ELOVL*) FAs. Further, *ELOVL6* preferentially utilizes SFA and MUFA as a substrate (e.g., C16:0 and C16:1*n*-7, respectively)^37^. To be noted, previous studies in Iberian × Landrace pigs (BC1_LD) provided evidence of the link between a polymorphism in the promoter region of *ELOVL6* (*ELOVL6*:c.-533C > T) and the percentage of C16:0 and C16:1*n*-7 FAs in LD muscle and backfat^38^. In addition, the *ELOVL6*:c.-394G > A polymorphism was suggested as the causal mutation for the QTL on pig chromosome 8 (SSC8) that affects FA composition in BC1_LD pigs^39^. Despite this, our findings did not detect association between *ELOVL6* and SFA, and just a suggestive association with C18:1*n*-9 and the MUFA/PUFA ratio (Supplementary Table S1).

Among the genes with lipolytic effect, we detected malate dehydrogenase 1 (*MDH1*) associated to C18:1*n*-9, MUFA, and C18:2*n*-6/C18:3*n*-3 and ω6/ω3 ratios (Fig. 3). *MDH1* encodes an enzyme which catalyzes reversible oxidation of malate to oxaloacetate in many metabolic pathways (including the citric acid cycle), utilizing the NAD/NADH-dependent system (GeneCards ID: GC02P063557)^36^. The function of *MDH1* is primarily related to the production of aerobic energy for muscle contraction^40^, and it was reported as candidate gene of meat quality traits in Chinese pig breeds^41^. The expression of another gene, lipoprotein lipase (*LPL*), was negatively associated with C18:1*n*-7 (Fig. 3). *LPL* belongs to the PPAR signaling pathway (Fig. 2), and has the dual functions of triglyceride hydrolase and ligand/bridging factor for receptor-mediated lipoprotein uptake (GeneCards ID: GC08P019901)^36^. Therefore, *LPL* hydrolyzes circulating triglyceride containing chylomicrons and very low-density lipoproteins to produce free FA. These free FAs can be assimilated by different tissues, such as muscle and adipose tissue^42^.

Regarding the genes involved in lipogenesis, we detected sterol-C5-desaturase (*SC5D*) as positively associated with C20:2*n*-6, and with C18:2*n*-6/C18:3*n*-3 and ω6/ω3 ratios (Fig. 3). *SC5D* (also known as *SC5DL*) encodes an enzyme of cholesterol biosynthesis (GeneCards ID: GC11P121292)^36^. *SC5DL* has been found to be one of the downregulated genes related to cholesterol metabolism in various tissues (including muscle) of lambs^43^. In a similar way, 3-hydroxy-3-methylglutaryl-CoA reductase (*HMGCR*) was positively associated with C18:2*n*-6/C18:3*n*-3 ratio (Fig. 3). *HMGCR* is a rate-limiting enzyme in cholesterol synthesis (GeneCards ID: GC05P075336)^36^. *HMGCR* is also related to several biological processes including pyruvate dehydrogenase activity, coenzyme metabolic process and signal transduction. A previous report in a Duroc population provided evidence of the links between the expression of *HMGCR* gene in muscle with several traits such as carcass lean percentage, C18:0 and C18:2*n*-6 contents, but also showed a positive correlation with cholesterol-related traits, intramuscular fat (IMF), and C18:1*n*-9 and C16:0 FA content^44^.

Our results also reported the association of acyl-CoA synthetase long chain family member 1 (*ACSL1)* and glutamate oxaloacetate transaminase 1 (*GOT1*) genes with C18:2*n*-6/C18:3*n*-3 and ω6/ω3 ratios (Fig. 3). *ACSL1* participate in the long-chain fatty acid import and signal transduction biological process (Fig. 2). The protein encoded by *ACSL1* is an isozyme of the long-chain fatty-acid-coenzyme A ligase family (GeneCards ID: GC04M184755)^36^. *ACSL1* is involved in the synthesis of long-chain acyl-CoA esters, FA degradation and phospholipid remodeling^45^. Likewise, this gene was found to be associated with lipid metabolism and mitochondrial oxidation of FAs in pigs^46^. In relation to FA degradation, Zhou et al.^47^, suggested that *GOT1* was crucial for providing oxaloacetate at low glucose levels, likely to maintain the redox homeostasis. In our results, *GOT1* was also related to organonitrogen compound biosynthetic process and signal transduction GO terms (Fig. 2).

### Genes related to carbohydrate and lipid metabolism

We also identified several genes belonging to carbohydrate and lipid metabolism that were associated with various of the analyzed traits such as *PLIN1, CYCS, SFRP5, LEP* and *PPP1R1B* (Fig. 3). Among the well-known genes, we observed perilipin 1 (*PLIN1*) positively correlated to MUFA traits and metabolic ratios (MUFA/SFA and MUFA/PUFA), and negatively correlated with C20:2*n*-6 and C18:3*n*-3 traits (Fig. 3). *PLIN1* was also enriched in the PPAR signaling pathway (Fig. 2). *PLIN1* codifies for a protein belonging to the family of perilipins, which play a role in regulating intracellular lipid storage and mobilization^48^. Association between the expression of *PLIN1* and porcine IMF deposition and adipocyte differentiation has already been eported^49^. In addition, a lipolytic function of *PLIN1* and increased expression in subcutaneous adipose biopsies from Iberian pigs fed a carbohydrate-enriched diet have been reported^50^. In our results, the expression of cytochrome c somatic (*CYCS)* was negatively correlated with MUFA and positively with correlated PUFA. *CYCS* encodes a small *heme* protein that functions as a central component of the electron transport chain in mitochondria (GeneCards ID: GC07M025118)^36^.

The expression of leptin (*LEP*) was significantly correlated with more than one trait (Fig. 3) (i.e., positively correlated with MUFA traits, C16:1*n*-7/C16:0 and C18:1*n*-9/C18:0 ratios, and metabolic ratios as MUFA/SFA and MUFA/PUFA), but it was negatively with C18:1*n*-7/C16:1*n*-7 ratio. *LEP* encodes a protein that plays a major role in the regulation of energy homeostasis (GeneCards ID: GC07P128241)^36^. In addition, as a pleiotropic adipocytokine it can regulate several physiologic functions. *LEP* constitutes a circulating hormone that orchestrates behavioral and metabolic responses to nutrient intake^51^. Furthermore, *LEP* interacts with other hormonal mediators, regulators of energy status and metabolism (e.g., insulin or glucagon) to regulate growth and reproduction processes^52^. Indeed, *LEP* can regulates complex biological effects through its receptors. By using the Iberian pig as translational model for studies on metabolic syndrome, type 2 diabetes and nutrition-associated diseases, a polymorphism of the leptin receptor (LEPR c.1987C > T) has been informed to increase insatiability and obesity (i.e., state that in human medicine is called as leptin resistance)^53^, and as such, the Iberian pigs would be resistant to leptin-induced lipolysis^50^. However, in our study the *LEPR* gene was not associated with any FA traits.

In the present work, we observed the transferrin receptor (*TFRC)* as negatively correlated with C18:1*n*-9 and MUFA traits, but positively correlated with C20:2*n*-6 and C18:2*n*-6/C18:3*n*-3 ratio (Fig. 3). *TFRC* (also known as *TFR1)* encodes a cell surface receptor necessary for cellular iron uptake by the process of receptor-mediated endocytosis (GeneCards ID: GC03M196027)^36^. In mammals, *TFR1* imports the transferrin-bound iron from the extracellular environment into cells. Interestingly, the intracellular labile free iron is indispensable for lipid peroxidation and ferroptosis execution^54^. On the other hand, protein phosphatase 1 regulatory inhibitor subunit 1b (*PPP1R1B*) was positively associated with MUFA traits but negatively correlated with PUFA traits (Fig. 3). *PPP1R1B* (also known as *DARPP-32)* encode a bifunctional signal transduction molecule, while stimulation of dopaminergic and glutamatergic receptors regulates its phosphorylation and function (GeneCards ID: GC17P039626)^36^. This gene is a potent inhibitor of protein phosphatase 1 (PPP1, previously known as PP1) when phosphorylated at Thr34 by cAMP-dependent protein kinase (PKA)^55^, and this protein could be important in the control of glycogen metabolism and muscle contraction, among other activities. Thus, it suggests that phosphorylation sites are implicated in the fine regulation of *DARPP-32* function and a modulation of the regulation of *PPP1* via *DARPP-32*. In addition, *PPP1R1B* has been reported as differentially abundant in the LD muscle of phenotypically extreme pigs^13^, and positively correlated with the IMF content of LD muscle in Duroc × Luchuan pigs^56^.

### FA and glucose transport genes

Five out of the 57 candidate genes were functional classified as FA and glucose transporters. Regarding FA transporters, our results suggested a significant association of four members of the solute carrier (SLC) gene superfamily with FA ratios and C20:2*n*-6 (Fig. 3). Members of SLC superfamily encode membrane-bound transporters, which play essential roles in transporting a variety of substrates across cellular membranes. These include amino acids, glucose, inorganic cations and anions, FAs and lipids, acetyl coenzyme A, and vitamins, among others^57^. Out of all the SLC members detected, the solute carrier family 27 member 1 (*SLC27A1)* has also been reported as a transporter of the predominant substrates of long-chain FAs^58^. Regarding glucose transporter, *ENSSSCG00000017801* was identified among the novel genes reported in our study, which was significantly associated with MUFA and PUFA traits, and metabolic ratios (Fig. 3). However, *ENSSSCG00000017801* is a novel gene recently annotated in pigs and thus, there is limited information in the literature about its functions. Nevertheless, the human orthologous of this novel gene is the trafficking regulator of GLUT4 1 (*TRARG1*), which is predicted to be involved in endosome to plasma membrane protein transport and glucose import in response to insulin stimulus (NCBI Gene ID: 286,753)^59^. Likewise, *TRARG1* is of particular interest as it was previously recognized to be located in the glucose transporter type 4 (*GLUT4*) storage vesicles, and to positively regulate *GLUT4* trafficking or translocation^60^.

### Regulators including transcription factor and co-factors

In order to reinforce the biological significance of our study, we also focused the discussion on regulators including those TFs and co-factors found among the list of candidate genes. Ladybird homeobox 1 (*LBX1*) was positively associated with C18:1*n*-9 and MUFA (Fig. 3). *LBX1* is a member of the ladybird-like gene family which encodes a homeodomain transcription factor^61^. *LBX1* plays a putative regulatory role during the postnatal development of the porcine skeletal muscle in Meishan pigs^62^. In the present study, we also observed association between well-documented co-factors of FA metabolism. Two of such genes were the nuclear receptor coactivator 2 (*NCOA2*) and lipin 1 (*LPIN1*), which were positively associated with ω6/ω3 ratio (Fig. 3). *NCOA2* encodes a protein that acts as a transcriptional coactivator for nuclear hormone receptors, including steroid, thyroid, retinoid, and vitamin D*.* In fact, a key role of *NCOA2* as modulator of the intramuscular FA composition in pigs has been reported^63^. On the other hand, *LPIN1* encodes a regulatory enzyme that catalyzes the penultimate step in triglyceride synthesis, including the dephosphorylation of phosphatidic acid to yield diacylglycerol (GeneCards ID: GC02P011677)^36^. He et al.^64^ reported a link between a polymorphism located in *LPIN1* gene with the percentage of leaf fat and IMF in pigs.

Another interesting co-factor was the galectin-related inhibitor of proliferation (*LGALS12*). We observed pleiotropic associations of *LGALS12*, which was positively associated with C16:1*n*-7, C18:1*n*-9, MUFA, MUFA/SFA, C16:1*n*-7/C:16:0 and C18:1*n*-9/C18:0, but negatively correlated with C20:2*n*-6 (Fig. 3). This gene participates in signal transduction and regulation of cellular process (Fig. 2). *LGALS12* was found to perform a critical role in lipid metabolism in mice, functioning as an intrinsic negative regulator of lipolysis, regulating lipolytic protein kinase A signaling by acting upstream of phosphodiesterase activity to control cAMP levels^65^. Lastly, Wu et al.^66^ reported that *LGALS12* knockdown could inhibit adipogenesis in porcine adipocytes by downregulating lipogenic genes and activating the PKA–Erk1/2 signaling pathway.

## Conclusions

Taken together, our results identify candidate genes linked to intramuscular FA composition in muscle, including well-known and novel candidate genes involved in biological processes and pathways mainly related to energy, lipid, and carbohydrate metabolism that appear to be determinant in the modulation of intramuscular FA profile in pigs.

## Supplementary Information

Supplementary Legends.

Supplementary Table S1.

Supplementary Table S2.

Supplementary Table S3.

Supplementary Table S4.

## Data Availability

All relevant data produced or evaluated in this research are disclosed in the paper as well as its supplementary information files. The RNA sequencing data used and analyzed in the current study are available from sequence read archive (SRA), NCBI BioProject under the accession number PRJNA882638 (https://www.ncbi.nlm.nih.gov/sra).
