# Supplementary material for: Global analysis of the association between pig muscle fatty acid composition and gene expression using RNA-Seq
Source: Sci Rep. 2023 Jan 11;13:535. doi: 10.1038/s41598-022-27016-x (PMC9834388; doi:10.1038/s41598-022-27016-x)
Supplement: Supplementary file 1 — Supplementary Legends. [file 41598_2022_27016_MOESM1_ESM.docx]

**Supplementary information**

**Supplementary table legends**

**Supplementary Table S1.** List of associated genes per FA traits in *LD* muscle. This association analysis was performed with ELMSeq approach. The beta coefficients (β1, β2 and β3) are estimates of the equation parameters corresponding to the gene expression, sex and slaughterhouse batch variables, respectively. With the *P*-value calculated for each gene, these raw values were corrected by the Benjamini and Hochberg procedure, and a threshold < 0.05 was used to determine significantly associated genes.

**Supplementary Table S2.** Total number of genes associated with FA traits in *LD* muscle. The identification of the type and name of gene was performed using BioMart tool. Each column with a gene name indicates that the gene was found within that label, which is available in our manually elaborated list from our knowledge base on functional annotation and previous publications. While novelty column was generated by geneshot tool.

**Supplementary Table S3.** Functional analysis with and without “GO Term Fusion” using ClueGO plugin in Cytoscape. In each sheet are available all GO terms significantly over-represented according to the FA group and their respective list of associated genes.

**Supplementary Table S4.** Pearson correlations between phenotypic values of FAs and gene expression normalized according to the ELMSeq step. The relationship between variables is illustrated through the correlation coefficient and the *P*-value columns. The BH adjusted *P*-value column indicates values according to the Benjamini and Hochberg procedure.
